# Who is Tracking Health on Mobile Devices: Behavioral Logfile Analysis in Hong Kong

**DOI:** 10.2196/13679

**Published:** 2019-05-23

**Authors:** Lu Guan, Tai-Quan Peng, Jonathan JH Zhu

**Affiliations:** 1 Department of Media and Communication City University of Hong Kong Hong Kong China (Hong Kong); 2 Department of Communication Michigan State University East Lansing, MI United States; 3 School of Data Science City University of Hong Kong Hong Kong China (Hong Kong)

**Keywords:** mobile apps, mHealth, circadian rhythm

## Abstract

**Background:**

Health apps on mobile devices provide an unprecedented opportunity for ordinary people to develop social connections revolving around health issues. With increasing penetration of mobile devices and well-recorded behavioral data on such devices, it is desirable to employ digital traces on mobile devices rather than self-reported measures to capture the behavioral patterns underlying the use of mobile health (mHealth) apps in a more direct and valid way.

**Objective:**

The objectives of this study were to (1) assess the demographic predictors of the adoption of mHealth apps; (2) investigate the temporal pattern underlying the use of mHealth apps; and (3) explore the impacts of demographic variables, temporal features, and app genres on the use of mHealth apps.

**Methods:**

Logfile data of mobile devices were collected from a representative panel of about 2500 users in Hong Kong. Users’ mHealth app activities were analyzed. We first conducted a binary logistic regression analysis to uncover demographic predictors of users’ adoption status. Then we utilized a multilevel negative binomial regression to examine the impacts of demographic characteristics, temporal features, and app genres on mHealth app use.

**Results:**

It was found that 27.5% of mobile device users in Hong Kong adopt at least one genre of mHealth app. Adopters of mHealth apps tend to be female and better educated. However, demographic characteristics did not showcase the predictive powers on the use of mHealth apps, except for the gender effect (B_female_ vs B_male_=–0.18; *P*=.006). The use of mHealth apps demonstrates a significant temporal pattern, which is found to be moderately active during daytime and intensifying at weekends and at night. Such temporal patterns in mHealth apps use are moderated by individuals’ demographic characteristics. Finally, demographic characteristics were also found to condition the use of different genres of mHealth apps.

**Conclusions:**

Our findings suggest the importance of dynamic perspective in understanding users’ mHealth app activities. mHealth app developers should consider more the demographic differences in temporal patterns of mHealth apps in the development of mHealth apps. Furthermore, our research also contributes to the promotion of mHealth apps by emphasizing the differences of usage needs for various groups of users.

## Introduction

### Background

Mobile technologies, including wireless devices and sensors that are intended to be worn, carried, or accessed by users during normal daily activities [1], have brought significant transformations to many practices in public health. In particular, because of *always-on, always-on you* [2] quality of mobile technologies, health apps on mobile devices (hereinafter labeled “mHealth apps”) have provide unprecedented opportunities for ordinary people to develop social connections revolving around health issues, seek health-related information, track daily workouts, manage chronic health conditions, and make medical appointments [3,4]. mHealth apps are found to play important roles in changing users’ health behaviors such as smoking cessation, weight management, and physical activity [5-8].

Understanding the adoption and use of mHealth apps has become an increasingly important research question in public health research. According to a population-based survey in Germany in 2015, 21% of smartphone users adopt mHealth apps [9]. Demographic factors are found to predict the adoption and use of mHealth apps. Users of mHealth apps tend to be younger, better educated, and in more healthy conditions [10]. However, previous research on mHealth apps was dominated by self-reported measures in surveys or interviews, in which participants were solicited to report their use of mHealth apps and other health-related behaviors. It has been widely acknowledged that the validity of self-reported measurements is threatened by many confounding factors such as memory errors, intentional distortion, cognitive factors, and social context factors [11]. Individuals may underestimate their health-risk behaviors to avoid the pressure of breaking social norms, whereas they may overestimate their health-conducive behaviors to meet social desirability.

With well-recorded behavioral data on mobile devices, it is desirable to employ digital traces on mobile devices rather than self-reported measures to capture behavioral patterns underlying the use of mHealth apps in a more direct and valid way. More importantly, the time-stamped digital traces can empower researchers to understand the temporal patterns, such as circadian rhythms and weekday-weekend difference, in the use of mHealth apps. The circadian rhythm in human behavior plays a crucial role in guiding people’s daily lives [12]. Empirical studies have found that maintaining the biological circadian rhythm is a necessity for human health [13]. Users of mHealth apps, who have relatively high health consciousness and health literacy [14], are expected to follow certain circadian rhythm in their use of mHealth apps. However, the fine-grained temporal information is extremely difficult, if not impossible, to be garnered in traditional self-reported survey research. The time-stamped behavioral data on mobile devices provide an unprecedented opportunity to understand how and when individuals will use mHealth apps.

Hong Kong provides a very good testbed to understand the adoption and use of mHealth apps. Hong Kong is one of the most populous cities in the world but also among the top 10 regions with the greatest life expectancy at birth [15]. Mobile cellular and the internet have been widely adopted in Hong Kong, whose penetration rates were 250% for mobile cellular by 2017 [16] and 87% for the internet by 2018 [17]. Among all internet users in Hong Kong, 98% of them go on the internet via mobile devices.

### Objectives

By analyzing the behavioral log data of mobile devices collected from a representative panel of ordinary users in Hong Kong, we aim to (1) assess the demographic predictors of the adoption of mHealth apps, (2) investigate the temporal pattern underlying the use of mHealth apps, and (3) disentangle the intertwined effects of demographic factors, temporal features, and app genres on the use of mHealth apps. The findings of this study advance our knowledge of demographic profiles of the adopters of mHealth apps and enhance our understanding of behavioral patterns underlying the use of mHealth apps, which possesses significant implications for promoting the adoption and use of mHealth apps in different dimensions of public health.

## Methods

### Data Collection

The data of the study were collected from a mobile audience study in Hong Kong. Users in the panel were recruited by a marketing research firm for local media organizations. To assure the representativeness of the panel sample, the panel was weighted against Hong Kong population census estimates in terms of the cross-distribution of age and gender. An on-device meter was installed on mobile devices (using Android and iPhone) of the users in the sample, to passively track all the activities on the devices without interrupting the users, who were informed about and agreed to the tracking and subsequent analyses of mobile app uses under the anonymous condition. The starting and ending time of each app use was recorded by the installed meter. The observation period for this study was from July to November 2017. All personally identifiable information about the users was removed before we were given access to the data. To further protect user privacy, the specific name of all mobile apps was removed, although the generic category (eg, Communication, News, Health, Games) is available, which provides the basis for this study to measure mHealth app use.

The apps adopted by all users were categorized into dozens of genres based on Google mobile app categories. Only the adoption and use of health apps are examined in this study. Of all the apps adopted by the users, 155 are health apps, which are categorized into 5 genres based on their functional features: generic activity tracking apps (51/155, 32.9%), health records log apps (36/155, 23.2%), sleep management and relaxation apps (29/155, 18.7%), training and coaching apps (23/155, 14.8%), and weight and diet management apps (16/155, 10.3%).

### Data Analysis

To understand who is more likely to adopt mHealth apps, a binary logistic regression is run to predict the adoption status of mHealth apps among mobile device users, with adopters of mHealth apps coded as 1 and nonadopters coded as 0. This analysis includes the whole panel of all users (N=2,591). Demographic variables, including gender, age, education degree, occupation status, parenting status, and marital status, are included as explanatory variables in the model.

Furthermore, following Dutton et al’s conceptualization of patterns of use for technologies [18], the use of mHealth apps is operationalized as the duration of each app use, which is calculated as the time lag between the start time and end time of each app use. To understand the temporal patterns underlying the use of mHealth apps, 2 temporal features are constructed based on the temporal information of each app use. First, the hour of a day when an mHealth app is triggered is recoded into 5 time windows on a 24-hour cycle: morning (6-10), noon (11-13), afternoon (14-17), evening (18-21), and night (22-5). Second, the weekday for the occurrence of an mHealth app use is also recoded into a binary variable with weekday (ie, Monday to Friday) being coded as 0 and weekends (ie, Saturday and Sunday) being coded as 1.

Then, a multilevel negative binomial regression is run to account for within-individual and between-individual level differences on the use of mHealth apps. This analysis only includes participants who adopted the mHealth apps (N=713). Negative binomial regression is employed here to handle the dependent variable with discrete probability distributions, such as count data and time data [19]. As the use records under the same user could be influenced by the unique characteristics of that particular user, a multilevel design is employed to unravel the within-individual and between-individual variations in the use of mHealth apps. Specifically, users’ demographic characteristics are included as independent variables at the between-individual level, whereas the 2 constructed temporal features (ie, daily time windows and weekday-weekend) and the genres of mHealth apps are included as independent variables at the within-individual level.

## Results

### Descriptive Statistics

Table 1 shows the descriptive statistics of demographic variables for all users in the panel. The average age of all users in the panel is 34 years, and 47.20% (1223/2591) are women. Among 2591 users in the panel, 40.80% (1057/2591) have high education level (ie, college degree or above), 20.57% (533/2591) medium education level (ie, associate degree), and 38.52% (998/2591) low education level (ie, high school education or lower). Users in the panel work as managers, administrators, or professionals (746/2591, 28.79%); clerks (640/2591, 24.70%); workers (482/2591, 18.60%); and students (396/2591, 15.28%), whereas 12.50% (583/2591) are unemployed (including housewives and retired). A total of 41.10% (1065/2591) are married, and 33.19% (860/2591) have at least 1 kid at home.

It was found that 27.52% (713/2591) of the users in the study adopt at least one genre of mHealth apps. The majority of the users (578/713, 81.1%) adopt only 1 genre of mHealth apps, 15.0% (107/713) adopt 2 genres, and 3.8% (27/713) adopt at least 3 genres.

The most popular genre of mHealth apps is the generic activity tracking, followed by health records log apps, weight and diet management apps, training and coaching apps, and sleep management and relaxation apps. On average, users spend 85.8 seconds in 1-time health app usage (SD=238.2 seconds). Users spend the longest time on sleep management and relaxation apps (mean=160.8 seconds, SD=435 seconds), followed by health records log apps (mean=94.8 seconds, SD=261 seconds), training and coaching apps (mean=91.8 seconds, SD=208 seconds), generic activity tracking (mean=79.8 seconds, SD=220 seconds), and training and coaching apps (mean=70.8 seconds, SD=169.8 seconds).

### Who Adopts Mobile Health Apps?

The binary logistic regression analysis results are summarized in Table 2. Female users are more likely to adopt mHealth apps than males (odds ratio, OR=1.44; *P*<.001). Better-educated users are more likely to adopt mHealth apps than less-educated users. Clerks are more likely to adopt mHealth apps, in comparison with workers (OR=1.68; *P*<.001). However, age, marital status, and parenting status of mobile device users do not significantly influence the adoption of mHealth apps.

**Table 1:** Descriptive statistics of demographic variables (N=2591).

Demographic variables		Frequency, n (%)
**Gender**		
	Male	1368 (52.80)
	Female	1223 (47.20)
**Age group (years)**		
	18-30	1164 (44.92)
	31-45	993 (38.32)
	46-64	434 (16.75)
**Education**		
	Low education level	998 (38.52)
	Medium education level	534 (20.61)
	High education level	1059 (10.87)
**Occupation**		
	Managers, administrators, and professionals	746 (28.79)
	Clerks	641 (24.74)
	Workers	482 (18.60)
	Students	397 (15.32)
	Unemployed	325 (12.54)
**Parenting status**		
	With kids at home	1730 (66.77)
	Without kids	861 (33.23)
**Marital status**		
	Married	1525 (58.86)
	Single, divorced or widowed	1066 (41.14)

**Table 2:** Binary logistic regression coefficients predicting the adoption of mobile health apps^a^ (N=2591).

Independent variables		Odds ratio (95% CI)	*P* value
Female (vs male)		1.44 (1.20-1.73)	<.001
Age (years)		0.99 (0.98-1.00)	.09
**Education (vs low education level)**			
	Medium education level	1.34 (1.04-1.71)	.02
	High education level	1.45 (1.16-1.81)	<.001
**Occupation (vs workers)**			
	Managers, administrators, and professionals	1.27 (0.94-1.72)	.12
	Clerks	1.69 (1.27-2.24)	.02
	Students	1.17 (0.83-1.65)	.37
	Unemployed	1.21 (0.86-1.69)	.28
Parenting status (with kids at home=1, without kids=0)		0.95 (0.77-1.18)	.66
Marital status (married=1, single/divorced/widowed=0)		1.24 (0.98-1.57)	.07

^a^Cox and Snell *R*^2^=0.022.

### What Accounts for the Duration of Mobile Health App Use?

A null model with the intercept only is first estimated to get the intraclass correlation coefficient (ICC). The ICC is 0.36, indicating that 36% of the variance in the use of mHealth apps could be attributed to between-individual differences (eg, the demographic characteristics of individuals). The high ICC detected here also implies that a multilevel design can provide adequate statistical power to account for the use of mHealth apps. A series of 3 multilevel negative binomial regressions are run to uncover the between-individual and within-individual antecedents underlying the use of mHealth apps. The analytical results of main effects are summarized in the Multimedia Appendix 1.

The conditional *R*^2^ is estimated for the 3 multilevel models reported in Multimedia Appendix 1. The conditional *R*^2^ describes the proportion of variance explained by both the between-level and within-individual factors included in the 3 models. Model 1, which includes the between-individual variables as independent variables, explains 35.6% of the variance in the use of mHealth apps. The addition of within-individual factors in model 2 increases the explanatory power by 0.8% (*P*<.001; likelihood-ratio, LR χ^2^_9=_ 145). Model 3, which incorporates the interactions between within-level factors (ie, daily time window, weekday-weekend, and health app genre) and between-individual factors, adds 1.1% of the variance (*P*<.001; LR χ^2^_90=_ 609).

Gender is the only significant demographic predictor of use of mHealth apps. Female users (estimated duration=80.2 seconds, 95% CI [72.9-87.6]) tend to spend less time on mHealth apps than male users (estimated duration=96.1 seconds, 95% CI [86.3-105.9]). It is surprising to find that all other between-individual variables, including age, education degree, occupation status, parenting status, and marital status, do not exert statistically significant main effects on the use of mHealth apps.

Temporal patterns have significant effects on the use of mHealth apps. Users are found to spend more time on mHealth apps during weekends (estimated duration=88.3 seconds) than during weekdays (estimated duration=85.2 seconds). Meanwhile, the use of mHealth apps tends to be more enduring at night (estimated duration=93.2 seconds), whereas less enduring in the morning (estimated duration=82.9 seconds).

There are significant differences among the use of different genres of mHealth apps. Training and coaching apps is the most enduring (estimated duration=126 seconds), followed by sleep management and relaxation apps (estimated duration=110 seconds), health records log apps (estimated duration=95.2 seconds), weight and diet management apps (estimated duration=90.2 seconds), and generic activity tracking apps (estimated duration=79.3 seconds).

The temporal patterns in the use of mHealth apps are found to be moderated by between-individual variables, as suggested by the significant cross-level interaction effects between temporal features, app genres, and demographic characteristics.

**Figure 1:**
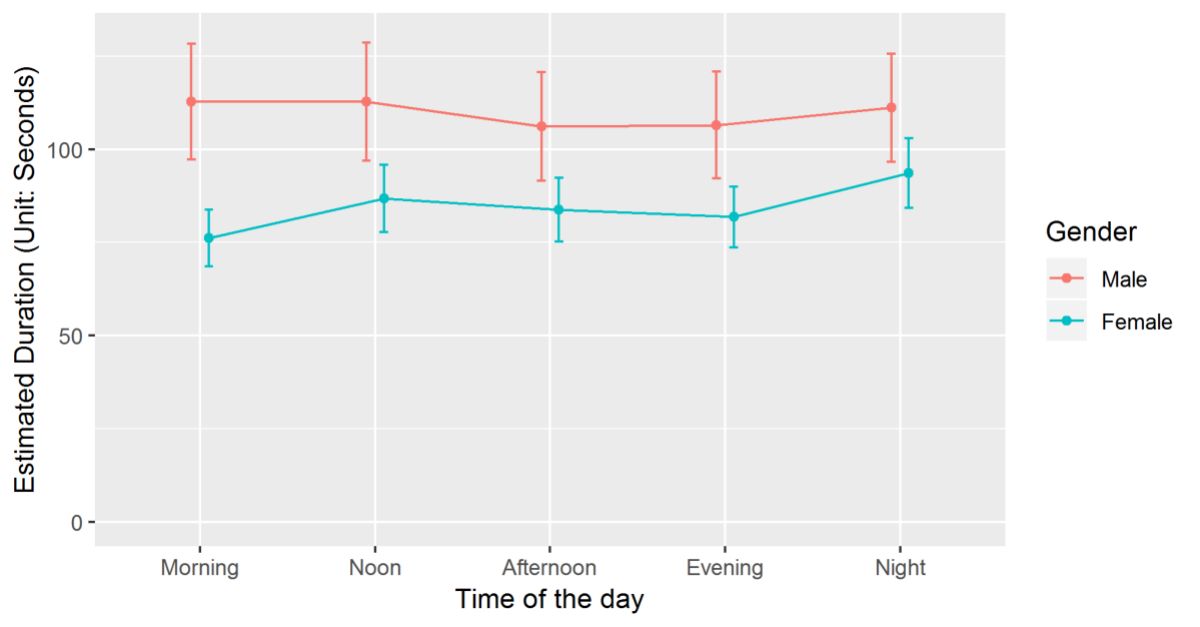
Interaction effect of gender and time window of the day on the length of use of mobile health apps.

First, the gender difference on the use of mHealth apps is significantly greater in the morning than that in other time windows of the day, as shown in Figure 1. Second, users in different age groups demonstrate different temporal patterns in the use of mHealth apps. Users of different age groups spend significantly similar amount of time on mHealth apps at noon and night, whereas older users spend less time on mHealth apps in the morning and evening compared with the youngsters.

Third, users’ education level is found to condition the temporal patterns in the use of mHealth apps. Users with different education levels tend to spend similar amount of time on mHealth apps in the morning and at night, whereas better-educated users spend significantly less time on mHealth apps in the daytime and during the weekends.

Besides, occupation is found to moderate the temporal patterns in the use of mHealth apps. Users of different occupations spend similar amount of time on mHealth apps at noon and night, whereas in the afternoon and evening time, housewives and retired users spend notably more time, and students spend significantly less time. Besides, student and unemployed users are found to have more stable use time schedules on mHealth apps no matter it is weekday or weekend, whereas workers, managers, administrators, and professionals spend significantly less time on mHealth apps during the weekdays than weekends.

Finally, our work also observed that demographics could differ the use of different genres of mHealth apps. First, the gender difference on the use of sleep management and relaxation apps is significantly greater than that of generic activity tracking apps. Second, education level is found to moderate the use of different genres of mHealth apps. Users with different education levels tend to spend similar amount of time on the use of generic activity tracking apps, whereas users with middle-level education spend significantly less time on training and coaching apps.

**Figure 2:**
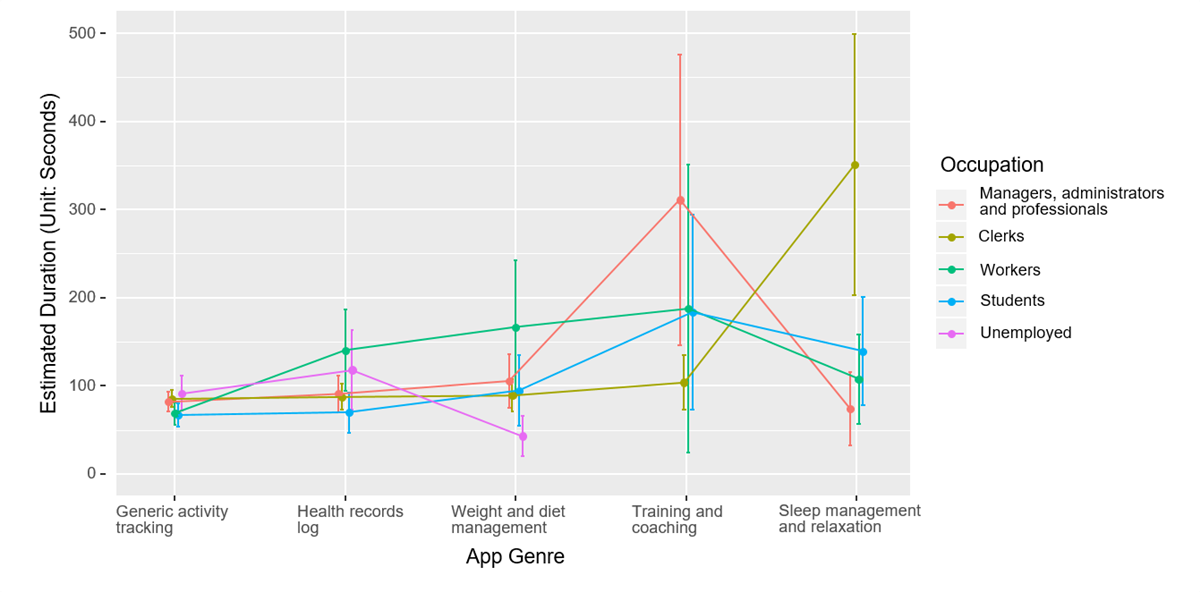
Interaction effect of occupation and app genre on the length of use of mobile health apps.

As shown in Figure 2, users’ occupation is found to condition the use of different genres of mHealth apps. Managers, administrators, and professionals spend longer time on training and coaching apps and less time on sleep management and relaxation apps. Clerks are found to spend the longest time on sleep management and relaxation apps among all the occupations.

Parenting and marital status are also found to be moderate the use of different genres of mHealth apps. The differences between users with different parenting status are larger in the use of training and coaching apps compared with the use of generic activity tracking apps, whereas the differences between users with different marital status are larger in the use of health records log apps and sleep management apps.

## Discussion

### Principal Findings

The first objective of this study was to assess the demographic predictors of health app adoption and use with a valid logfile dataset. Our results are consistent with previous studies that better educated and female mobile device users are more likely to adopt mHealth apps. However, we did not find the significant main effect of other demographic variables on the use of mHealth app, except for the gender difference. Second, our study aims to investigate the temporal pattern of health app use. The results indicate that the use of mHealth apps not only demonstrates a circadian rhythm but also differentiates between the weekdays and weekends. Noon and night are the peaks for the use of mHealth apps around a day. Besides, users spend longer time on mHealth apps on weekends than on weekdays. Finally, the findings show significant interaction effects between temporal features, app genres, and demographic characteristics on the use of mHealth apps. The gender difference on the use of mHealth apps is significantly greater in the morning. Users of different age groups spend significantly similar amount of time on mHealth apps at noon and night, whereas older users spend less time on mHealth apps in the morning and evening compared with the youngers. Besides, better-educated users spend significantly less time on mHealth apps in the daytime and during the weekends. We also found that demographics (gender, education, occupation, parenting status, and marital status) could differ the use of different genres of mHealth apps.

### Comparison With Prior Work

Compared with prior studies, the adoption rate of mHealth apps in Hong Kong revealed in this study is quite higher than that found in Germany [10] and the United States [14] and that found in Hong Kong in 2016 [20]. Consistent with prior research [9,10], our work shows that females with better education background are more likely to adopt a health app. The consistency indicates a relatively high validity of self-report measures on health app adoption. However, our results show some discrepancy with prior studies on the use of mHealth apps. Only gender is found to play a role in explaining the mHealth app use in our study. However, we did not find the significant main effects of other demographic variables (eg, age and education) as claimed in previous studies. This is possibly because of the difference of the measurements. Prior studies measured the app use as individual’s self-reported frequency of use, whereas our study aims to measure it in a more correct way, as calculating the actual duration of app use. As individuals may overestimate their health activity frequency to meet the social desirability, the validity of self-reported measure is often questioned especially in the frequency estimates [21]. Further efforts are needed to verify our results and re-examine the demographic effects in terms of the degree of health app use.

The study also finds a weekly and daily circadian rhythm of mHealth apps use. People spend longer time on health apps on the weekends than in a weekday. Besides, health app use reaches the peak at noon and night, throughout the 24-hour cycle a day. The pattern is largely matched with the temporal pattern in the use of mobile devices that mobile phone use being moderately active during the daytime but intensifying during break time and after work time [22,23]. These could be explained by the time availability theory that one of the most powerful predictors of technology use is whether the users have available time at that certain moment [24,25]. People are more likely to use mHealth apps during the weekends and the break time at noon and night because they have more spare time to engage in mobile app activities.

Finally, our findings provide evidence that users of different demographic (ie, gender, age, education, occupation, and parenting status) demonstrate different temporal patterns in the use of mHealth apps across circadian rhythm and the day of the week. First, the use difference between male and female in the morning indicates the gender role in the household: wives generally take more housework and child care responsibilities at home in the morning [26]. The unequal division of household workload responds to the gender differences of time allocation on health-related activities. Second, our result suggests that family role could also influence the temporal patterns in the use of mHealth apps for users with different parenting status. Users who have kids at home spend less time on mHealth apps during the weekends than the weekdays, which is exactly inversed with the users without kids. This is possibly because the workload for parents increases dramatically when their children come back home on the weekends. Besides, our work indicates that users of different occupations have different time schedules in the use of mHealth apps. The differences may reflect the outcomes of the social division of labor. Time in paid labor could constraint user’s time allocation on other activities (eg, household time, leisure time, and health-related activity time) [27]. Thus, the employed and unemployed share distinct circadian clock patterns in the use of mHealth apps. Finally, our findings provide evidences for educational and age differences in the temporal patterns of the use of mHealth apps. The reasons are unclear but may be related to the social norms and cohort effects in health habit formation.

### Limitations and Future Work

This study is subject to some limitations that can be addressed in future studies. For example, we lack the information about user’s health conditions, initial motives, and behavioral outcomes in the use of mHealth apps. Research has found strong predictive powers of motivations on health-related behavior change (eg, HIV prevention and smoking cessation) [28]. People with different health conditions and motivations may also demonstrate different temporal patterns in the use of mHealth apps, which may further lead to different behavioral change outcomes. For example, people with stronger motivations may have more stable use schedules no matter the time window of the day or the day of the week, which may positively influence the health-related behavioral change. Future studies could consider combining the digital track data with survey or interview approaches to investigate the cognitive and biological mechanism underlying the health-related behavioral change in terms of the temporal patterns.

Second, although the meter in this study could passively track the activities on the devices without any interruptions to the panelists, there is concern that surveillance may have effects on user’s behaviors on mobile phone. One possible assumption is that panelists may avoid doing extreme activities (eg, watching adult movies) and try to conduct more health-conducive activities to meet social desirability. However, this limitation should have minimal impacts on the validity of the results, as the efforts for panelists to take to meet the social desirability in this study are relatively high (eg, users have to participate in real training and coaching activities for several months). This impact could be rather minimal compared with that of self-reported measures because participants in survey method could easily conceal their real status and report more frequent health activities without any actual efforts.

### Conclusions

By analyzing the behavioral log data of mobile devices collected from a representative panel in Hong Kong, this study explores the temporal patterns in the use of mHealth apps and examines the intertwined effects of demographic factors, temporal features, and app genres on the use of mHealth apps. Users of different demographic characteristics are found to have their own preference of the app genres and distinct schedules on app use across the circadian rhythm and the week.

Our study could contribute to the public health research and industries both theoretically and practically. First, our research adds to existing research by reporting the temporal patterns of mHealth app use. Maintaining the biological circadian rhythm is a necessity for human health. The intervention of the circadian rhythm in an individual’s health-related habit could influence their health situation. Thus, understanding the temporal patterns of individual’s health app use is of great importance for health maintaining and health intervention research.

Second, our study could contribute to the promotion of health-related apps and activities. Our work shows that users with different demographic characteristics prefer different genres of mHealth apps. For example, managers, administrators, and professionals spend more time on training and coaching apps, whereas less time on sleep management and relaxation apps. Thus, our findings can assist the health promotion practitioner accurately select the appropriate target group and meet the health needs of the target audience.

Finally, our research could also contribute to the development of mHealth apps. We explored the temporal patterns in the use of mHealth apps and found that users of different demographic (ie, gender, age, education, occupation, and parenting status) have different use schedules on mHealth apps across the circadian rhythm and the week. Thus, mHealth app developers could consider more about the demographic differences in temporal patterns when they design and develop health apps to meet the customers’ needs.

## Appendix

Multimedia Appendix 1 Multilevel negative binomial regression predicting the use of mobile health apps.

## Abbreviations

ICC: intraclass correlation coefficient
LR: likelihood-ratio
mHealth: mobile health
OR: odds ratio
