# Supplementary material for: Who is Tracking Health on Mobile Devices: Behavioral Logfile Analysis in Hong Kong
Source: JMIR Mhealth Uhealth. 2019 May 23;7(5):e13679. doi: 10.2196/13679 (PMC6552450; doi:10.2196/13679)
Supplement: Multimedia Appendix 1 [file mhealth_v7i5e13679_app1.pdf]

Multimedia Appendix 1

This is a Multimedia Appendix to a full manuscript published in the J Med Internet Res. For full copyright and citation information see http://dx.doi.org/10.2196/jmir.13679

Who is Tracking Health on Mobile Devices: Behavioral Logfile Analysis in Hong Kong

Lu Guan[1], MA; Tai-Quan Peng[2*], PhD; Jonathan JH Zhu[1,3*], PhD

[1]Department of Media and Communication, City University of Hong Kong, Hong Kong, China (Hong Kong)
[2]Department of Communication, Michigan State University, East Lansing, MI, United States
[3]School of Data Science, City University of Hong Kong, Hong Kong, China (Hong Kong)
[*] these authors contributed equally

Corresponding author:
Tai-Quan Peng, PhD
Department of Communication
Michigan State University
404 Wilson Road Room 473
East Lansing, MI, 48824
United States
Tel: (+1) 517-355-0221
Email: winsonpeng@gmail.com

Table 1. Multilevel negative binomial regression predicting the use of mobile health apps[a b c d].

Variable	Model 1		Model 2		Full model	
	Estimate (95% CI)	*P* value	Estimate (95% CI)	*P* value	Estimate (95% CI)	*P* value
**Between-individual variables**						
Female (vs male)	–0.135 (–0.264 to –0.007)	.04	–0.180 (–0.309 to –0.052)	.006	–0.112 (–0.268 to 0.044)	.16
Age	–0.004 (–0.012 to 0.005)	.40	–0.003 (–0.011 to 0.006)	.55	0.002 (–0.008 to 0.012)	.74
**Education (vs low education level)**						
Medium education level	–0.090 (–0.269 to 0.09)	.33	–0.090 (–0.268 to 0.088)	.32	0.081 (–0.132 to 0.294)	.46

High education level	–0.071 (–0.234 to 0.093)	.40	–0.072 (–0.234 to 0.090)	.38	–0.024 (–0.212 to 0.164)	.80
**Occupation (vs workers)**						
Managers, administrators, and professionals	–0.043 (–0.265 to 0.180)	.71	–0.026 (–0.246 to 0.195)	.82	0.039 (–0.233 to 0.311)	.78
Clerks	0.024 (–0.182 to 0.229)	.82	0.033 (–0.171 to 0.237)	.75	0.124 (–0.130 to 0.378)	.34
Students	–0.087 (–0.342 to 0.168)	.51	–0.116 (–0. 369 to 0. 137)	.37	–0.121 (–0.437 to 0.195)	.45
Unemployed	0.119 (–0.133 to 0.370)	.36	0.139 (–0.110 to 0.388)	.27	0.098 (–0.233 to 0.428)	.56
Parenting status (with kids at home=1, without kids=0)	0.073 (–0.078 to 0.225)	.34	0.064 (–0.086 to 0.214)	.40	–0.009 (–0.193 to 0.176)	.93
Marital status (married=1, single, divorce or widow=0)	–0.114 (–0.287 to 0.059)	.20	–0.127 (–0.299 to 0.045)	.15	–0.195 (–0.409 to 0.018)	.07
**Within-individual variables**						
Weekends (vs weekdays)	—	—	0.031 (0.005 to 0.056)	.02	0.218 (0.040 to 0.396)	.02
**Time window of day (vs noon)**						
Morning	—	—	–0.076 (–0.118 to –0.035)	<.001	–0.021 (–0.320 to 0.278)	.891
Afternoon	—	—	–0.044 (–0.086 to 0.001)	.04	–.151 (–0.456 to 0.154)	.332
Evening	—	—	–0.059 (–0.099 to –0.019)	.004	–0.056 (–0.35 to 0.237)	.708

		Night	—	—	0.042 (0.001 to 0.083)	.04	–0.355 (–0.643 to –0.067)	.02
	**App genres (vs generic activity tracking apps)**							
		Health records log apps	—	—	0.175 (0.086 to 0.264)	<.001	1.216 (0.421 to 2.012)	.003
		Weight and diet management apps	—	—	0.128 (0.037 to 0.218)	.006	1.503 (0.667 to 2.339)	<.001
		Training and coaching apps	—	—	0.481 (0.369 to 0.593)	<.001	2.152 (0.641 to 3.663)	.005
		Sleep management and relaxation apps	—	—	0.344 (0.211 to 0.477)	<.001	2.165 (0.797 to 3.533)	.002
**Interaction terms of variables**								
	**Time Window of Day * Gender**							
		Morning * female	—	—	—	—	-0.129 (-0.232 to -0.026)	.01
	**Time Window of Day * Age**							
		Morning * Age	—	—	—	—	-0.009 (-0.016 to -0.002)	.01
	**Time Window of Day * Education**							
		Morning * High Education Level	—	—	—	—	0.163 (0.051 to 0.275)	.004
		Night * High Education Level	—	—	—	—	0.154 (0.047 to 0.260)	.005
	**Time Window of Day * Occupation**							

		Morning * Clerks	—	—	—	—	0.19 (0.009, 0.371)	.04
		Morning * Unemployed	—	—	—	—	0.452 (0.176, 0.728)	.001
		Afternoon * Managers, administrators, and professionals	—	—	—	—	0.318 (0.130, 0.507)	.001
		Afternoon * Clerks	—	—	—	—	0.229 (0.045, 0.412)	.01
		Afternoon * Unemployed	—	—	—	—	0.389 (0.137, 0.642)	.003
		Evening * Unemployed	—	—	—	—	0.295 (0.047, 0.543)	.02
		Night * Students	—	—	—	—	0.239 (0.025, 0.452)	.03
	**Day of a Week * Education**							
		Weekends * High Education Level	—	—	—	—	-0.091 (-0.159, -0.022)	.01
	**Day of a Week * Occupation**							
		Weekends * Clerks	—	—	—	—	-0.172 (-0.279, -0.065)	.002
		Weekends * students	—	—	—	—	-0.181 (-0.317, -0.046)	.009
		Weekends * Unemployed	—	—	—	—	-0.269 (-0.43, -0.109)	.001

	**App Genres * Gender**						
		Sleep management and relaxation apps * female	—	—	—	—	-0.601 (-1.039, -0.163)	.007
	**App Genres * Education**						
		Health records log apps * Medium education level	—	—	—	—	-0.469 (-0.762, -0.177)	.002
		Weight and diet management * Medium education level	—	—	—	—	-0.791 (-1.13, -0.452)	<.001
		Weight and diet management * High education level	—	—	—	—	-0.753 (-1.038, -0.469)	<.001
		Weight and diet management * High education level	—	—	—	—	-1.597 (-2.384, -0.811)	<.001
		Training and coaching apps *	—	—	—	—	-0.644 (-1.17, -0.117)	.02

		High education level						
		Sleep management and relaxation apps * Medium education level	—	—	—	—	-1.316 (-2.01, -0.623)	<.001
	**App Genres * Occupation**							
		Health records log apps * Managers, administrators, and professionals	—	—	—	—	-0.611 (-0.998, -0.223)	.002
		Health records log apps * Clerks	—	—	—	—	-0.691 (-1.04, -0.343)	<.001
		Health records log apps * Students	—	—	—	—	-0.67 (-1.147, -0.193)	.006
		Weight and diet management * Managers, administrators, and professionals	—	—	—	—	-0.635 (-1.162, -0.108)	.02

		Weight and diet management * Clerks	—	—	—	—	-0.843 (-1.296, -0.389)	<.001
		Weight and diet management * Unemployed	—	—	—	—	-1.641 (-2.38, -0.902)	<.001
		Sleep management and relaxation apps * Clerks	—	—	—	—	0.964 (0.348, 1.581)	.002
		Sleep management and relaxation apps * Unemployed	—	—	—	—	3.832 (2.213, 5.451)	<.001
	**App Genres * Parenting Status**							
		Training and coaching apps * With kids at home	—	—	—	—	1.188 (0.726, 1.65)	<.001
		Sleep management and relaxation apps * With kids at home	—	—	—	—	0.627 (0.159, 1.096)	.009

**App Genres * Marital Status**							
	Health records log apps * Married	—	—	—	—	0.288 (0.023, 0.554)	.03
	Sleep management and relaxation apps * Married	—	—	—	—	-1.063 (-1.615, -0.511)	<.001
Constant		4.50 (4.16 to 4.83)	<.001	4.44 (4.11 to 4.78)	<.001	4.22 (3.80 to 4.64)	<.001
**Model summary**							
	Variance of intercepts (SD)	0.432 (0.657)	—	0.434 (0.033)	—	0.423 (0.650)	—
	Log-likelihood	–211,777	—	–211,704	—	–211,400	—
	Akaike information criterion	423,602	—	423,517	—	423,364	—
	Conditional R^2	0.356	—	0.364	—	0.375	—

[a] Following Dutton et al's conceptualization of patterns of use for technologies, the use of mobile health apps is operationalized as the duration of each app use, which is calculated as the time lag between the start time and end time of each app use.

[b] Model 1 only includes the between-individual variables as independent variables; model 2 adds within-individual variables; full model include both between-individual variables, within-individual variables, and interaction terms of variables. Thus, variables that are not included in model are filled with dashes.

[c] Number of use records=40035; Number of users=713.

[d] Only significant interaction terms were reported.
